# CD26 Expression on T-Anaplastic Large Cell Lymphoma (ALCL) Line Karpas 299 is associated with increased expression of Versican and MT1-MMP and enhanced adhesion

**DOI:** 10.1186/1471-2407-13-517

**Published:** 2013-11-01

**Authors:** Pamela A Havre, Long H Dang, Kei Ohnuma, Satoshi Iwata, Chikao Morimoto, Nam H Dang

**Affiliations:** 1 Division of Hematology/Oncology, University of Florida Shands Cancer Center, Gainesville, FL 32610, USA; 2 Department of Therapy Development and Innovation for Immune Disorders and Cancers, Graduate School of Medicine, Juntendo University, Tokyo 113-8421, Japan; 3 Division of Hematology/Oncology, University of Florida, 1600 SW Archer Road, Box 100278, Gainesville, Florida 32610, USA

**Keywords:** CD26, T-cell malignancies, Adhesion, MT1-MMP, Cell signaling

## Abstract

**Background:**

CD26/dipeptidyl peptidase IV (DPPIV) is a multifunctional membrane protein with a key role in T-cell biology and also serves as a marker of aggressive cancers, including T-cell malignancies.

**Methods:**

Versican expression was measured by real-time RT-PCR and Western blots. Gene silencing of versican in parental Karpas 299 cells was performed using transduction-ready viral particles. The effect of versican depletion on surface expression of MT1-MMP was monitored by flow cytometry and surface biotinylation. CD44 secretion/cleavage and ERK (1/2) activation was followed by Western blotting. Collagenase I activity was measured by a live cell assay and in vesicles using a liquid-phase assay. Adhesion to collagen I was quantified by an MTS assay.

**Results:**

Versican expression was down-regulated in CD26-depleted Karpas 299 cells compared to the parental T-ALCL Karpas 299 cells. Knock down of versican in the parental Karpas 299 cells led to decreased MT1-MMP surface expression as well as decreased CD44 expression and secretion of the cleaved form of CD44. Parental Karpas 299 cells also exhibited higher collagenase I activity and greater adhesion to collagenase I than CD26-knockdown or versican-knockdown cells. ERK activation was also highest in parental Karpas 299 cells compared to CD26-knockdown or versican-knockdown clones.

**Conclusions:**

Our data indicate that CD26 has a key role in cell adhesion and invasion, and potentially in tumorigenesis of T-cell lines, through its association with molecules and signal transduction pathways integral to these processes.

## Background

CD26/dipeptidyl peptidase IV (DPPIV) is a 110–115 kD glycosylated protein that exists as a homodimer. It is a multifunctional membrane protein with three domains: extracellular, transmembrane, and cytoplasmic. It is widely expressed on a number of tissues and can regulate tumor growth and development [1-7]. The interaction of CD26/DPPIV with other proteins, including collagen, fibronectin, and caveolin-1, likely influences its involvement in cell motility and invasion [8,9]. CD26 and its associated DPPIV enzyme activity play a key role in T-cell biology, serving as a marker of T-cell activation and participating in several signaling pathways [10-13]. CD26 is also a marker of aggressive cancers, including T-cell malignancies [14-20]. Interestingly, the cleaved form of CD26, which is present in plasma, is inversely correlated with several aggressive cancers [21].

Our previous work showed that CD26-depleted human T-anaplastic large cell lymphoma (T-ALCL) Karpas 299 cells were unable to form tumors in SCID mice [8], and that CD26 expression on two T-cell lines increased SDF-1-α-mediated invasion [22]. We were interested in looking at CD26-associated gene products involved in cell motility and therefore conducted microarray analysis of genes involved in this pathway in parental Karpas 299 and CD26-depleted clones, and found that versican expression was associated with changes in CD26 level*.* Microarray analysis revealed that mRNA level for versican was considerably lower in CD26-depleted Karpas 299 cells than parental Karpas 299 cells (1:88). Although mRNA levels for several other genes, including IGFBP3, tenascin C, and SPOCK1, were also lower in CD26-depleted cells than parental Karpas 299, Western blots confirmed a difference in protein expression for versican only, but not for the other three proteins. Versican is a large chondroitin sulfate proteoglycan involved in the regulation of adhesion, migration, invasion, and angiogenesis [23]. Versican binds to ECM constituents including type I collagen, fibronectin, and hyaluronan (HA) [24] and a number of cell-surface proteins, including CD44, integrin β1, and toll receptor 2 [25,26]. Versican levels are elevated in most malignancies, and correlated with poor patient outcome. Versican is secreted by peritumoral stromal cells and also by the individual cancer cells [27,28]. Four major isoforms exist that differ with respect to the number and position of GAG molecules attached, which are important for association with other proteins. Of note is that the V0 and V1 isoforms are reported to be the isoforms most closely associated with cancers.

In the present paper, we examined in detail CD26 involvement with cell migration and adhesion in T-cell lines. Expression array analyses of genes involved in extracellular matrix and adhesion pathways indicated that versican expression was significantly higher in parental T-ALCL Karpas 299 cells compared to CD26-depleted Karpas 299 cells. To further investigate the relationship between CD26 and versican, we conducted knock down studies of versican in Karpas 299 cells and evaluated for a potential effect on expression of signaling proteins and adhesion. We found that the use of shRNA to knock down versican expression in the parental Karpas 299 cells resulted in both lower MT1-MMP transcription and surface expression. To confirm that cell behavior was consistent with the observed change in MT1-MMP activity, several assays were performed; secretion and cleavage of CD44, collagenase I activity, and adhesion. In all three assays, parental Karpas 299 cells exhibited higher activity compared to cells in which CD26 or versican was knocked down. Finally, ERK activation, which is required for migration and invasion, was also highest in the parental Karpas 299 cell line.

## Methods

### Reagents

Bovine serum albumin (BSA), polybrene (hexadimethrine bromide), sodium dodecyl sulfate, glycine, sodium deoxycholate, trypsin, phosphate buffered saline, and dimethyl sulfoxide were from Sigma Life Science, St. Louis, MO. TX-100, NP-40, and Tween-20 were from Fisher Scientific, USA. Puromycin was from Life Technologies, USA. Rat tail collagen and bovine skin collagen were purchased from BD and Advanced Matrix, respectively. GM6001, a general MMP inhibitor was purchased from Calbiochem.

### Cell culture

Karpas 299 cells were originally obtained from the American Type Culture Collection (ATCC, Manassas, VA) and maintained in RPMI-1640 (Hyclone, Logan, UT). Karpas 299 cells depleted of CD26 have been described previously [8]. All cell media contained 10% fetal bovine serum (Hyclone), penicillin (100 u/ml) and streptomycin (100 μg/ml).

### Expression arrays

GEArray express human extracellular matrix and adhesion molecule microarrays were carried out by SuperArray Bioscience Corporation on 10 μg total RNA isolated from parental Karpas 299 cells and Dep1, a cell line deficient in CD26 expression.

### Real-time RT-PCR

Real-time RT-PCR was carried out on 10 ng total RNA (RNeasy kit, Qiagen). SYBR Green-based real-time RT-PCR was carried out using QuantiTect Primer Assays (Qiagen) for CD26 (Hs_DPP4_1_SG), Versican (Hs_VCAN_1_SG), and GAPDH (Hs_GAPDH_1_SG).

### RT-PCR

RT-PCR was carried out on 10 ng of RNA isolated from parental Karpas 299 cells, Dep1, and Dep2 using the Titan One Tube RT-PCR system (Roche Applied Science). The primers were described previously [29]. The sizes of the amplification products were 405 bp for V0 (forward: 5′- TCAACATCTCATGTTCCTCCC-3′ and reverse: 5′-TTC TTCACTGTGGGTATAGGTCTA-3′) and 336 bp for V1 (forward: 5′-GGCTTTGACCAGTGC GATTAC-3′ and reverse: 5′-TTCTTCACTGTGGGTATAGGTCTA-3′). The reverse transcription step was carried out at 50° for 30 min, followed by denaturation for 2 min at 94°, amplified by 35 cycles (94° for 30 s, 55° for 45 s, 68° for 45 s) and elongated for 7 min at 68°.

### Flow cytometry

Cells were washed once with staining buffer (PBS containing 1% BSA) and incubated on ice for 30 minutes with antibodies specific for the activity domain of MT1-MMP (ab51074, Abcam, Cambridge, MA), then with FITC goat anti-rabbit Ig at 0.125 μg/10^6^ cells (BD Pharmingen). After washing with staining buffer twice, the cells were resuspended in PBS. The optimum amount of MT1-MMP antibody was determined by titration.

### Gene silencing

Transduction ready viral particles for gene silencing of versican (versican shRNA, Santa Cruz Biotechnology, Inc., Santa Cruz, CA) were used to infect Karpas cells at a ratio of 0.5 virus particles per cell. Cells were pelleted the following day, resuspended in fresh media, and 48 hrs following transduction, puromycin was added at a concentration of 2.5 ug/ml. Following selection, stable clones were isolated by limiting dilution. Knockdown was monitored by running whole cell lysates and/or spent media on gels and probing with versican antibodies as described in the Western Blot section.

### Cell lysis

Cells were lysed using RIPA (1% NP40, 0.5% DOC, 0.1% SDS, 150 mM NaCl, 50 mM TrisCl, pH 8.0) or TX100 buffer (50 mM TrisCl, pH 8, 0.15 M NaCl, 1% TX-100) containing a protease/phosphatase inhibitor cocktail (Pierce, Rockford, IL). Protein concentration was determined using the bicinchoninic acid protein assay reagent (Pierce).

### Isolation of vesicles from serum free media

Cells (8 × 10^6^) were grown in serum free media for 48 hours, followed by centrifugation at 600 ×g for 15 min, then 1500 × g for 15 min, and the resulting supernatant was subsequently centrifuged at 100,000 × g for 1 hr at 4°C. Pelleted vesicles were suspended in PBS and assayed for protein [30].

### Western blots

Equal amounts of protein were run on 5.0, 7.5% or 10% polyacrylamide gels. For detection of versican, samples were combined with sample buffer without reducing agent. Following transfer, blots were blocked, then probed with one of the following antibodies: anti-CD26 (AF1180) and anti-CD44H (clone 2C5) were from R & D Systems, Inc., Minneapolis, MN; anti-versican (clone 2B1, Seikagaku, Tokyo, Japan); and anti-MT1-MMP (ab38971, Abcam). Anti-phospho-p44/42 MAPK (Erk ½) and anti-p44/42 MAPK (Erk ½) were from Cell Signaling Technology, Inc; anti-integrin alpha 5 chain (BD, cat# 610633). Precision Plus Protein Standards (Bio-Rad Laboratories, Hercules, CA) were run to estimate sizes of proteins of interest. Horseradish peroxidase-conjugated secondary antibodies and the detection reagent, SuperSignal West Dura Extended Duration Substrate, were from Pierce. Films were scanned using an Image Quant 400 (GE Healthcare, Piscataway, NJ).

### Biotinylation and immunoprecipitation

Cells were suspended in PBS (2.5 × 107/ml) and incubated with 200 μl of 10 mM EZ-Link^®^ Sulfo-NHS-LC-Biotin/ml cells for 30 min on ice. The cells were then washed 3× with PBS containing 100 mM glycine. Following lysis in TX100 buffer, 1 mg lysate was applied to a Streptavidin- Agarose spin column (Pierce), and following extensive washing, bound proteins were eluted with 2× sample buffer and heating at 100°C for 5 min. Eluates were run on 7.5% acrylamide gels and probed with anti-MT1-MMP antibody.

### Collagen degradation in cultured cells

Collagen I degradation was monitored in live cells migrating through a native 3D collagen substrate. DQ™ collagen, type I from bovine skin, fluorescein conjugate (Molecular Probes) was copolymerized with rat-tail collagen type I, in RPMI media without phenol red (Life Technologies). After incubation for 48 hrs at 37°C, solid phase collagen and cells were pelleted and the supernatant analyzed for FITC using a Perkin-Elmer Victor^3^ V multilabel counter [31].

### Collagen degradation in vesicles

The EnzChek collagenase assay (Life Technologies) was used to evaluate activity in vesicles isolated from conditioned media. In this assay, DQ™ collagen, type I from bovine skin, fluorescein conjugate (Molecular Probes) was used as substrate and the incubation was carried out at room temperature as described by the manufacturer. Each well of a 96 well plate contained 4.5 μg vesicle protein. Fluorescence was detected using the Perkin-Elmer instrument.

### Adhesion assays

Adhesion assays were carried out essentially as described [8]. Cells (5 × 10^5^/well) were seeded into 12 well collagen I coated plates and incubated overnight. Unattached cells were removed, plates were washed three times with PBS and the adhesive cells remaining were quantified using the MTS assay. The total cell number was determined using uncoated wells and serial dilutions were used to construct a standard curve to convert absorbance at 490 nm to cell number.

## Results

### Model showing idealized scheme for interaction of signaling molecules in parental Karpas 299 cells

**Figure 1:**
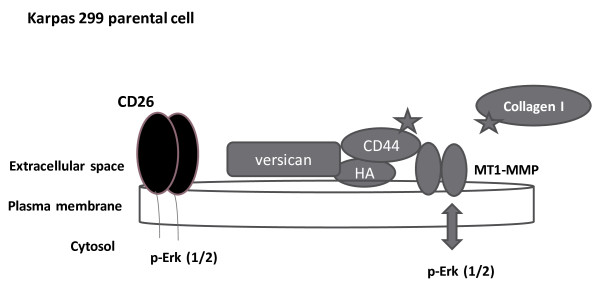
**Model for CD26 regulation of adhesion and downstream signaling.** In this simplified scheme, CD26 is shown bound to the cell membrane. Versican is also depicted in the membrane, but is also secreted and is a constituent of the extracellular matrix. CD44 and HA are bound to versican, but CD44 is also bound to MT1-MMP, which can itself cleave CD44, resulting in CD44 secretion. Secretion of the cleaved CD44 is necessary for localization of MT1-MMP at the invadopodia where it digests collagen I, a constituent of the extracellular matrix. In addition, Erk (1/2) activation occurs in the parental Karpas cells and has been reported to be required for migration, invasion, and CD44 upregulation. This model is intended to be a working hypothesis of the relationship between the proteins shown here.

Figure 1 depicts a simplified scheme for molecules believed to be involved in CD26 enhanced invasion. In this proposed model for parental Karpas 299 cells, CD26 is shown bound to the cell membrane. Results from our microarray analysis indicated that in CD26-depleted cells, versican was underexpressed, at a ratio of 1:80 compared to the parental cell. Versican is an extracellular matrix component and is involved in diverse activities, including adhesion, proliferation, migration, and angiogenesis. MT1-MMP is a membrane MMP and is also involved in these activities. It is one of the few MMPs that can degrade directly collagen I, a component of the extracellular matrix. CD44 binds to both versican and MT1-MMP, which is able to cleave CD44. It is thought that cleavage and release of CD44 from the membrane is required for the relocalization of MT1-MMP to the invadopodia, where it binds to collagen I, leading to invasion of the extracellular matrix. Relocation to the invadosome may occur in vesicles (or exosomes). Activation of Erk (1/2) is also shown here, since it is reported to form a positive feedback loop with MT1-MMP and has been shown to regulate invasive activity.

### Decreased expression of versican is associated with CD26 depletion in human T-anaplastic large cell lymphoma Karpas 299

Our previous work showed that depletion of CD26 in Karpas 299 cells resulted in loss of cell adhesion to the extracellular matrix and decreased tumorigenicity in a SCID mouse xenograft model [8]. To identify CD26-associated gene products potentially involved in cell adhesion processes, we performed expression microarray analysis of human extracellular matrix and adhesion molecules with RNA isolated from parental Karpas 299 and the CD26-depleted Karpas 299 cell line Dep1 [8]. Our data indicated that expression of versican was approximately 90-fold higher in the parental Karpas 299 cells compared to CD26-depleted Karpas 299 cells (Table 1).

**Table 1:** Oligo GE Array microarrays indicate that versican mRNA expression is higher in CD26-expressing cells than in CD26-depleted cells (Dep1)

**Unigene**	**RefSeqNo**	**Symbol**	**Dep1**	**Karpas**	**Karpas/Dep1**
Hs.544577	NM_002046	GAPDH	253.7	141.5	0.56
Hs.443681	NM_004385	VCAN	0.68	60.12	88.4

GEArray express human extracellular matrix and adhesion molecule microarrays were carried out by SuperArray Bioscience Corporation on 10 μg total RNA isolated from parental Karpas 299 cells and Dep1, a cell line deficient in CD26 expression.

**Figure 2:**
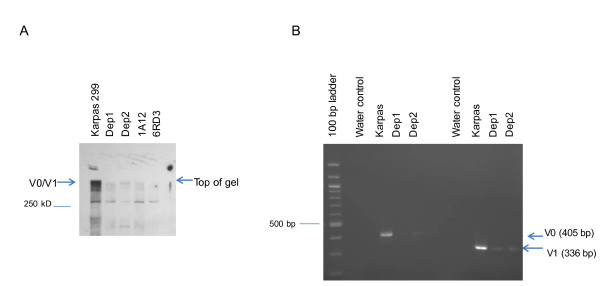
**Confirmation of Versican expression in Karpas 299 cells and in CD26-depleted and Versican-depleted Karpas cells. A**. Western blots confirmed versican expression in Karpas cell lines and clones resulting from knockdown of versican in parental Karpas 299 cells using shRNA. Whole cell lysates (30 μg) of Karpas, Dep1, Dep2, and two clones derived from knock down of versican in parental Karpas cells, 1A12 and 6RD3 were run on 7.5% gels. The top of the gel and 250 kD marker are indicated. Blots were probed with anti-versican antibody at 1:100 dilution, followed by anti-mouse HRP at 1:10,000 dilution. **B**. RT-PCR using V0 and V1 specific primers show product was present when RNA from the parental Karpas 299 cells was used but barely detectable when RNA from Dep1 or Dep2 was used as the template. Results from Western blots and RT-PCR were obtained from two independent experiments.

Real-time RT-PCR and Western blots were subsequently carried out to confirm differential expression of versican in parental Karpas 299 cells and the two CD26-depleted Karpas 299 cell lines Dep1 and Dep2 [8]. RNA was isolated from Karpas 299, Dep1, and Dep2 cells, and SYBR Green based real-time RT-PCR was performed using QuantiTect Primer Assays. Down-regulation of versican was confirmed in CD26 depleted cells, with an 80-fold and 103-fold enrichment for parental Karpas 299 compared to Dep1 and Dep2, respectively (Table 2). Western blot analyses also confirmed that versican expression was higher in parental Karpas 299 as compared to Dep1 and Dep2 (Figure 2A). RT-PCR using V0 and V1 specific primers were used to confirm this as shown in Figure 2B.

**Table 2:** Real-time RT-PCR was used to confirm Versican expression

**GAPDH**	**Avg Ct**	**Karpas/Dep1**	**Karpas/Dep2**
Karpas	17.74	-	-
Dep1	16.70	0.49	-
Dep2	16.72	-	0.49
**CD26**			
Karpas	20.93	-	-
Dep1	23.95	8.11	-
Dep2	24.05	-	8.69
**Versican**			
Karpas	25.51	-	-
Dep1	31.83	80	-
Dep2	32.20	-	103

RNA was isolated from Karpas 299 cells and two clones, Dep1 and Dep2, in which CD26 is depleted. SYBR Green-based real-time RT-PCR was carried out on 10 ng total RNA using QuantiTect Primer Assays for CD26, Versican, and GAPDH.

### Enhanced expression of MT1-MMP is associated with CD26 and versican in Karpas 299

MT1-MMP (MMP14) plays a critical role in the process of cell motility and invasion, with its deletion in tumor cells resulting in the loss of both *in vitro* and *in vivo* invasive activity [32]. We therefore examined its status in parental Karpas 299 and the CD26-depleted Karpas 299 Dep1 and Dep2 cell lines. In addition, to further evaluate the effect of versican depletion in the T-ALCL Karpas 299 cell line independent of CD26 status, we established a number of versican knock down Karpas 299 lines, as described in Materials and Methods and shown in Figure 2.

**Figure 3:**
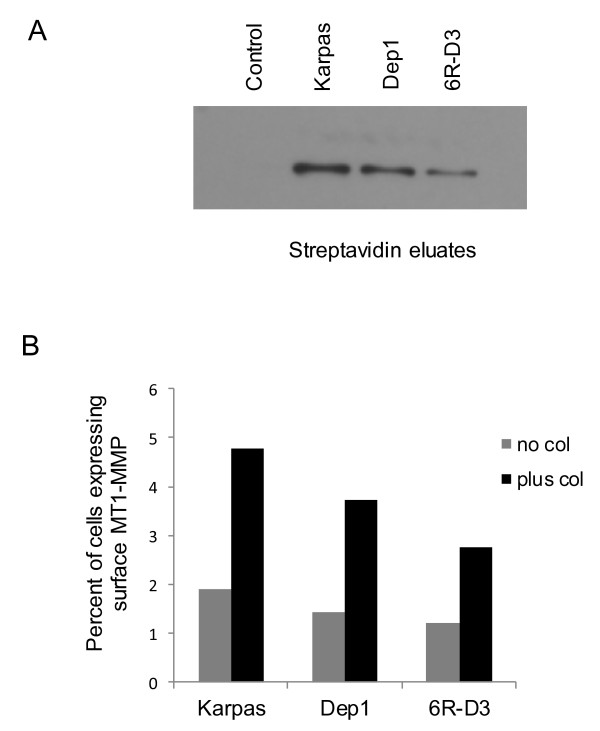
**Surface expression of MT1-MMP is higher in Karpas parental cells than in Dep1 (CD26 depleted) or 6RD3 (versican depleted). A**. Cells were grown overnight on collagen I plates, then biotinylated using an impermeable reagent. Lysates (1 mg protein) were applied to streptavidin-agarose spin columns, washed, and eluted with sample buffer. Eluates were run on 7.5% SDS gels, transferred to nitrocellulose, and probed with MT1-MMP antibodies. **B**. Flow cytometry of cells grown with and without collagen I. Data are representative of two independent experiments for panel **A** and for panel **B**.

Since only MT1-MMP expressed on the cell surface mediates degradation of the extracellular matrix [32], we next evaluated its surface expression by both cell surface biotinylation and flow cytometry analysis, as described in Materials and Methods. Cells were cultured overnight in collagen I coated wells to stimulate MT1-MMP expression [33]. Our data indicated that a higher percentage of parental Karpas 299 cells exhibited surface expression of MT1-MMP than CD26-depleted Dep1 or versican-knock down clone 6RD3 (Figure 3A).

Meanwhile, flow cytometry studies also demonstrated that the presence of collagen induced greater surface expression of MT1-MMP in all cells tested (Figure 3B). Importantly, a higher percentage of parental Karpas 299 cells expressed surface MT1-MMP than Dep1 or 6RD3 clones in the presence or absence of collagen. Of note is the fact that our experiments consistently found MT1-MMP to be expressed at relatively low levels on the cell surface, findings which were consistent with previous work demonstrating that only small amount of MT1-MMP is expressed on the cell surface at any one time [34].

### Enhanced CD44 expression is associated with CD26 and versican in Karpas 299

**Figure 4:**
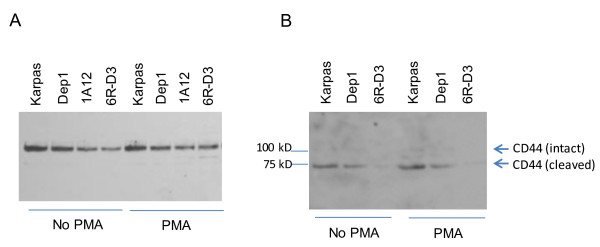
**CD44 expression/secretion of cleaved form is higher in parental Karpas 299 cells than in Dep1 or 6RD3 cells. A**. Whole cell lysates (30 μg) from cells grown on collagen I plates in the presence or absence of 10 ng/ml PMA for 24 hr. **B**. Concentrated conditioned media (75 μg) isolated from cells grown on collagen I plates for 24 hr. Samples were run on 7.5% SDS gels, transferred, and probed with anti-CD44H, followed by anti-mouse HRP. Of note is that intact CD44 migrates as a 100 kD protein, whereas the cleaved form migrates as a 70–75 kD species [36,67]. Data are representative of three independent experiments.

MT1-MMP has been reported to associate with several membrane-associated and cytosolic proteins, including CD44 [35]. Interaction of MT1-MMP with CD44 leads to the cleavage of CD44 and facilitates migration by indirectly linking MT1-MMP to the cytoskeleton [35,36]. Our present work demonstrated that expression of CD44 in total cell lysates (Figure 4A) and secretion of its cleaved form in conditioned media (Figure 4B) were higher in parental Karpas 299 as compared to the CD26-depleted Dep1 and versican-depleted 1A12 and 6RD3 clones. Since PMA has been shown to increase CD44 expression [37] and to stimulate trafficking of MT1-MMP to the plasma membrane [38-40], we conducted our studies in the presence or absence of PMA. In our experimental system, PMA had only a slight enhancing effect on the expression and secretion of CD44.

### Enhanced collagenase I activity is associated with CD26 and versican in Karpas 299 cells

**Figure 5:**
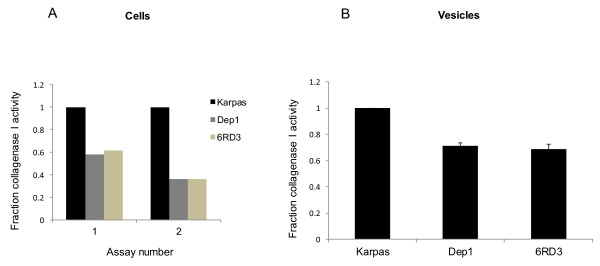
**Karpas 299 cells and vesicles exhibit greater collagenase I activity than either Dep1 or 6RD3 cells. A**. Collagen I degradation was monitored in live cells migrating through a native 3D collagen substrate. FITC-collagen type I from bovine skin was copolymerized with rat-tail collagen I. After 48 hr, cells and solid phase collagen were pelleted and the supernatant analyzed for FITC release. **B**. Collagen I degradation was also measured in vesicles isolated from conditioned media of cells grown for 48 hrs on collagen I. Two independent assays are shown for the intact cells **(A)** and three independent assays for the vesicles **(B)**. Error bars are standard error of the mean.

Previous work has demonstrated an association between MT1-MMP and enhanced collagen I degradation [32,41]. We next conducted two separate assays for collagenase I activity as described in Materials and Methods, one using a solid phase assay in which collagen I degradation was monitored in live cells (Figure 5A), and the other using a liquid-phase assay with vesicles isolated from conditioned media (Figure 5B). In both types of assays, parental Karpas 299 cells exhibited a higher level of collagenase I activity than Dep1 or 6RD3 clones.

### Adhesion to collagen I is highest in the parental Karpas 299 cell line

**Figure 6:**
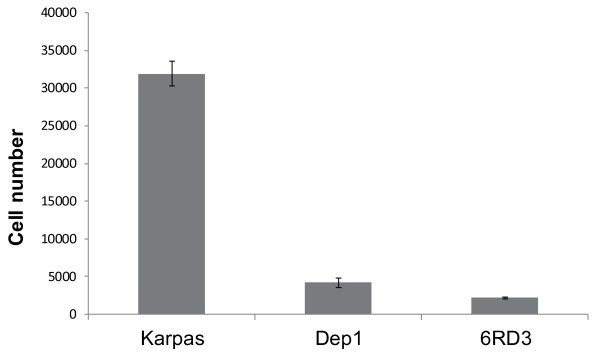
**Adhesion assays show that Karpas 299 cells adhere to collagen I to a greater extent than CD26-depleted, Dep1, or CD26-expressing, versican-depleted, 6RD3 cells.** Cells (5 x 10^5^/well) were seeded into 12 well collagen I coated plates and incubated overnight. Following removal of non-adhesive cells, the cells remaining were quantified using the MTS assay. The total cell number was determined using uncoated wells and serial dilutions were used to construct a standard curve to convert absorbance at 490 nm to cell number. Error bars are standard error of the mean. Data are representative of three independent experiments.

Adhesion to collagen I was compared for the parental Karpas 299 cells, the CD26-depleted cells (Dep1) and versican-depleted cells (6RD3) in precoated 12 well plates. Our findings indicated that the versican-expressing parental Karpas 299 cells exhibited much greater adhesion to collagen than the versican-depleted Dep1 and 6RD3 cell lines (Figure 6).

### Erk(1/2) activation is highest in the parental Karpas 299 cell line

**Figure 7:**
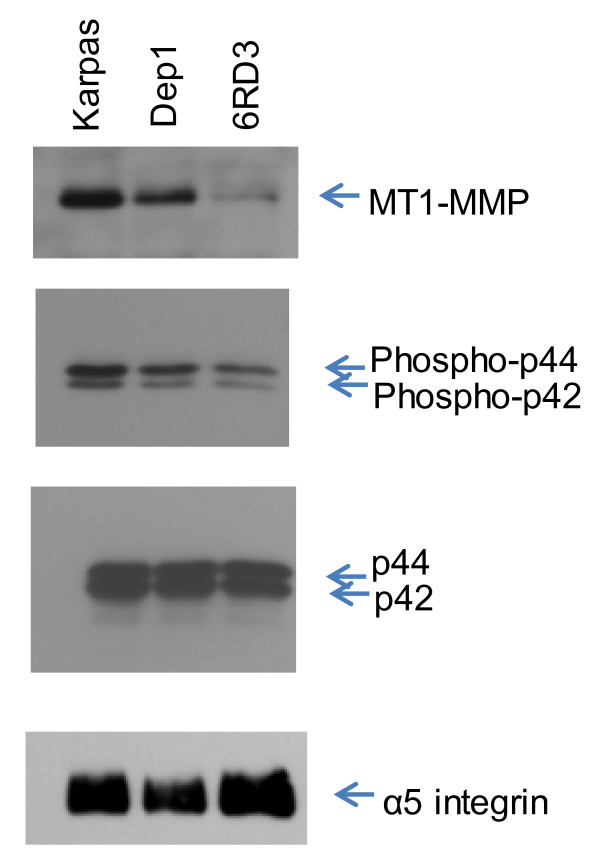
**Erk(1/2) activation is highest in the parental Karpas 299 cell line.** Cells (8 x 10^6^) were grown in serum free media for 48 hrs, centrifuged at low speed to remove cells and debris, then at 100,000 x g for 1 hr. Vesicles were suspended in PBS and assayed for protein. Equal amounts of protein (5 μg) were loaded in each well of a 7.5% SDS gel. Following transfer to nitrocellulose, blots were probed with anti-MT1-MMP antibody (top) or anti-phospho-p44/42 MAPK antibody (middle), stripped, and reprobed with anti-p44/42 MAPK antibody (next to bottom). The blot was also probed with anti-α5 integrin antibody (bottom). Data are representative of two independent experiments.

Erk (1/2) activation is required for CD44 [42,43] expression and cell migration and is induced by overexpression of MT1-MMP [44]. In addition, MT1-MMP expression activates Erk (1/2), which then leads to upregulation of MT1-MMP, creating a positive feedback loop [33]. To further explore the mechanism involved in MT1-MMP upregulation associated with CD26 and versican, cells were cultured overnight in serum free medium, and the expression of MT1-MMP, phosphorylated Erk (1/2), and integrin α5 in vesicles isolated from the conditioned medium was determined by Western blot (Figure 7). We had previously observed that activated Erk (1/2) and MT1-MMP were present in the conditioned media (data not shown) and others have shown that MT1-MMP is present in vesicles isolated from the spent media of endothelial [45], fibrosarcoma, and melanoma cells [46]. We found that the expression of MT1-MMP was higher in parental Karpas 299 cells than in the CD26-depleted Dep1 cells or versican-depleted 6RD3 cells. Activation of Erk (1/2) followed the same pattern, which is consistent with observations for actively migrating cells [38]. In contrast the level of the α5 integrin appeared to be similar in all cells.

## Discussion

In this paper, we have focused on the differential expression of versican in CD26-expressing Karpas 299 cells as compared to a CD26-depleted clone and the associated changes in various cellular activities as related to tumorigenesis. As a point of reference, we presented a working model at the beginning of the paper. The emphasis is placed on MT1-MMP (MMP-14), since it is known to have several important activities which could account for the ability of CD26-expressing Karpas 299 cells to form tumors in SCID mice as opposed to the inability of CD26-deficient Karpas 299 cells to develop tumors in the same animal model [8]. We do note that this simplified model does not take into account the complex roles that MT1-MMP and other MMPs play in cancer progression. For example, in addition to degrading the extracellular matrix, MT1-MMP plays an important role in tumor angiogenesis [47] through upregulation of VEGF [48] and immunoregulation through its effect on the release and activation of cytokines such as TGF-β, a well-known suppressor of T-lymphocyte reaction against cancer [49].

In addition to the difference in versican expression, there were differences in adhesion, MT1-MMP surface expression, CD44 cleavage and secretion, and collagenase I activity. Although CD26 is known to bind both collagen [50,51] and fibronectin [52], versican also binds these proteins, and can further strengthen the binding of CD26-expressing cells to the extracellular matrix. This conclusion is consistent with our observation that MT1-MMP surface expression was increased in cells bound to collagen I. Since localization of MT1-MMP to the cell membrane is required for its ability to degrade the extracellular matrix [32], the decreased surface expression of MT1-MMP associated with loss of versican would be predicted to have an effect on cell motility, and possibly, tumorigenesis by interfering with the ability of tumor cells to interact with the microenvironment.

Our present work also established a relationship between CD44, CD26 and versican, with CD44 cleavage/secretion being higher in parental Karpas 299 cells than in cells depleted of versican (both CD26-depleted cells as well as CD26-expressing/versican depleted cells). Interaction with and cleavage of CD44 by MT1-MMP has been shown to facilitate migration by indirectly linking MT1-MMP to the actin cytoskeleton [35,36]. The function of MT1-MMP is regulated in large part by its localization; MT1-MMP activity has been observed at invadopodia [53-55], lamellipodia [35], and focal adhesions [56], with CD44 cleavage and secretion appearing to play a role in the localization of MT1-MMP to the invadopodia [35].

Our data also indicated a higher level of ERK activation in parental Karpas 299 cells compared to CD26-depleted or CD26-expressiong/versican-depleted clones. ERK activation is required for migration, invasion [44,57,58], and CD44 upregulation. The requirement for matrix proteins along with ERK activation suggests that integrins may be involved in MT1-MMP regulation [59], a conclusion that is further supported by colocalization of integrins with MT1-MMP in vesicles [46,60] and the existence of common recycling pathways [61]. In a recent study, intracellular trafficking of MT1-MMP was found to be coupled with trafficking of integrin α5, ERK activation, and phosphorylation of MT1-MMP at Thr^567^[38]. We also detected these three proteins in vesicles isolated from conditioned media; MT1-MMP and phosphorylated ERK were highest in the parental Karpas 299 cells, whereas the amount of α5 integrin was approximately the same in all three cell lines.

Although regulation of versican expression is not well understood, it has been shown to be a target of Wnt signaling, regulated by the phosphatidylinositol 3-kinase (PI3K) pathway in human embryonic carcinoma cells [62]. It is possible that it is also regulated by this pathway in Karpas 299 cells, since activated Akt/PKB is higher in the parental Karpas 299 cells than in CD26-depleted or versican-depleted cells (unpublished observations, author).

In addition to its ability to form homodimers, CD26 can also form heterodimers with fibroblast activation protein alpha (FAP or Seprase) [63], which shares 48% homology with CD26 [64], but unlike CD26, can digest collagen. Although this protein complex has been detected at the invadopodia of migrating fibroblasts [65], we did not explore the role of Seprase activity in the collagenase I activity of Karpas 299 cells. However, our Western blot assays for Seprase did not detect a difference among parental Karpas 299 cells, Dep1, and 6RD3 (data not shown). While it has been suggested that CD26 and related proteins, such as FAP, may serve as valuable biomarkers for selected malignancies, better in-depth understanding of the functional roles of these molecules in particular tumor types and their associated microenvironment will improve our knowledge of the implications of their expression in tumor behavior [66].

## Conclusions

In summary, our data suggest that CD26 has a key role in cellular adhesion and invasion through versican and MT1-MMP expression as well as downstream signaling molecules involved in these processes. The expression of versican in Karpas 299 parental cells is likely responsible for their increased adhesion to the extracellular matrix, which is necessary for cellular interaction with ECM components and is also required for migration. The difference in the adhesiveness of the parental Karpas 299 cells and their CD26-deficient (and therefore versican deficient) counterpart, Dep1, may account for the difference in tumorigenicity previously observed in SCID mice [8].

## Competing interests

The authors declare that they have no competing interests.

## Authors’ contributions

PAH performed the research; PAH and NHD designed the research study, analyzed the data, and wrote the paper; KO, SI and CM contributed essential reagents and analyzed the data; LHD analyzed the data and critically revised the paper. All authors read and approved the final manuscript.

## Pre-publication history

The pre-publication history for this paper can be accessed here:

http://www.biomedcentral.com/1471-2407/13/517/prepub
